# Microbial fuel cell centric nutrient rebalancing and recycling from human waste in space missions

**DOI:** 10.1042/ETLS20240003

**Published:** 2025-09-11

**Authors:** Daniela Zertuche Moreno, Aradhana Singh, Dibyojyoty Nath, Ioannis A. Ieropoulos

**Affiliations:** Civil, Maritime & Environmental Engineering Department, School of Engineering, University of Southampton, SO16 7QF, U.K

**Keywords:** bioenergy, hydroponics, long-duration space mission, microbial fuel cells, nutrient recycling, urine treatment

## Abstract

Efficient human waste management and hygiene maintenance are vital for long-duration space missions. By using bioelectrochemical systems, specifically microbial fuel cells (MFCs) combined with hydroponics, human waste can potentially be converted into a valuable commodity. Recent advancements in MFCs indicate a significant potential for generating electricity (1–2 mW/single MFC/ml of urine) and biofertilisers concurrently from urine and sewage while suppressing human pathogens that may be present. Integrating MFCs with hydroponics opens up the possibility to balance nutrients in human waste while growing vegetables in MFC-powered hydroponic systems, using only a small percentage of synthetic fertilisers, if deemed necessary. This is a concise perspective of the potential of MFCs for nutrient recycling from human waste and vegetable production that could enhance the self-sustainability of a spacecraft or mission.

## Introduction

Long-term missions to establish surface habitats on the Moon and Mars are anticipated as early as 2030, based on data gathered from short-duration space missions and the International Space Station (ISS) [1,2]. Nevertheless, moving towards the next phase of human space exploration, where frequent resupply of consumables is infeasible, requires addressing an important gap in space technologies: ‘closed-loop systems’. The primary focus of these systems is not only on minimising consumables and resupply, but also on recovering desirable constituents from waste for subsequent use in food and energy production. For instance, bringing a year’s worth of meals for five crew members could add up to ~3300–4300 kg and ~3650 kg of food and water, respectively [3], making this an inefficient and costly solution for spacecraft storage and fuel. Thus, local food and energy production, alongside water recovery and waste management, are key processes for sustaining life in long-term surface habitats. This has pushed the scientific community in recent years to shift the waste-treatment perspective from ‘waste removal’ to ‘resource recovery’ [4]. Technologies in current development that can ‘close the loop’ must comply with the inherent challenges of long-term manned space missions, such as self-sustainability and robustness to operate intermittently in both zero- and micro-gravity environments, as well as being resilient, modular and scalable.

Long-term human missions will also result in producing significant amounts of urine, which are often managed with conventional techniques. On the other hand, urine can be profitably utilised with microbial fuel cells (MFCs), which are currently being assessed at large scale in certain well-established labs worldwide [5,6]. MFCs can use urine to produce electricity and recover high-value constituent components, facilitating elemental cycling. MFCs represent a way of profiting from the naturally occurring reactions of electroactive microorganisms that convert the chemical energy stored in biological waste into electricity. This is a short perspective of how MFCs can be well exploited in space missions by (i) harnessing urine for electricity generation and (ii) recycling elements for food production with an integrated MFC-hydroponics (MFCH) system.

## MFCs supporting the closed-loop model in space missions

### Conventional methods of urine treatment in space

During space missions, urine goes through the Urine Processor Assembly, where water content is recovered using vacuum compression distillation. The resulting urine brine, containing a smaller portion of water, is then passed through a Brine Processor Assembly containing a patented Ionomer-membrane Water Processor (IWP). The IWP consists of a first layer of microporous membrane and a second layer of ionomer membrane. The former allows bulk gas permeation, while the latter selectively allows water vapour to permeate, resulting in contained residual dehydrated brine and volatiles. The vapour is then condensed using the spacecraft’s heat exchanger(s) and processed into potable water by the Water Recovery System included in the space station’s Environmental Control and Life Support System [7]. While the current system in place has remarkable water recovery performance, valuable nutrients are lost, and a resupply of membranes is needed on a regular basis.

### MFCs for electricity generation from urine

For long-term missions, a closed-loop system, integrating MFCs as pre-treatment technology for nutrient recovery could be a favourable solution. MFCs can help ‘close the loop’ by generating fertiliser and/or recovering water *in situ* from waste treatment, simultaneously providing energy for the LEDs in plant cultivation as well as for general lighting purposes. While the design and configurations for MFCs could adapt to the desired application, the main elements of the MFCs remain the same: an anode chamber and a cathode chamber separated by a proton exchange membrane. Oxidative reactions occur in the anode chamber as a result of microbial metabolism of organic matter. As the substrate is biologically broken down, electrons flow through a whole range of biochemical conductive pathways, onto the anode electrode, before ending up on the cathode electrode, via a connecting external circuit, resulting in DC current production. Conversely, the reduction reaction happens in the cathode acting as an electron sink. The electrical output generated by the flow of electrons can then be used to power electronic devices. MFCs have proven to fully utilise a fresh urine sample of 25 ml in three days, within an individual MFC unit, with an absolute current output of 0.25 mA [8]. In the ISS, the urine and urinal flush liquids are calculated to be 1.5 kg per crew member per day [3]. Assuming a 2-l daily production of urine wastewater, 20 mA can be extracted from a single MFC [8]; the objective would naturally be to employ multiple MFC units configured into stacks for higher instantaneous current (power) output.

### Nutrient recovery and food production using integrated MFC-hydroponic (MFCH) system

More than 80% of the total nitrogen, phosphorus and potassium (NPK) content in a domestic wastewater stream is contained in human urine and faeces [9], depsite the fact that volumetrically, urine is a small fraction (<5%) of the total wastewater volume. Urine source isolation and element recovery have therefore been proven to be more successful strategies [10]. Urine can be treated concurrently with aiding in the production of power, particularly when connected to MFCs, as previously mentioned. Urine-fed ceramic cylindrical MFCs with internal air cathodes have been shown to achieve a maximum absolute power of up to 2.1 mW per unit. When operated under load, nutrient-rich catholyte (N, P, K along with other micronutrients) in these MFCs, promoted by electro-osmotic forces, has shown antimicrobial properties that inhibit human pathogens [11,12]. The catholyte was then used as a liquid fertiliser to grow basil (*Ocimum basilicum*) without any microbial contamination.

**Figure 1:**
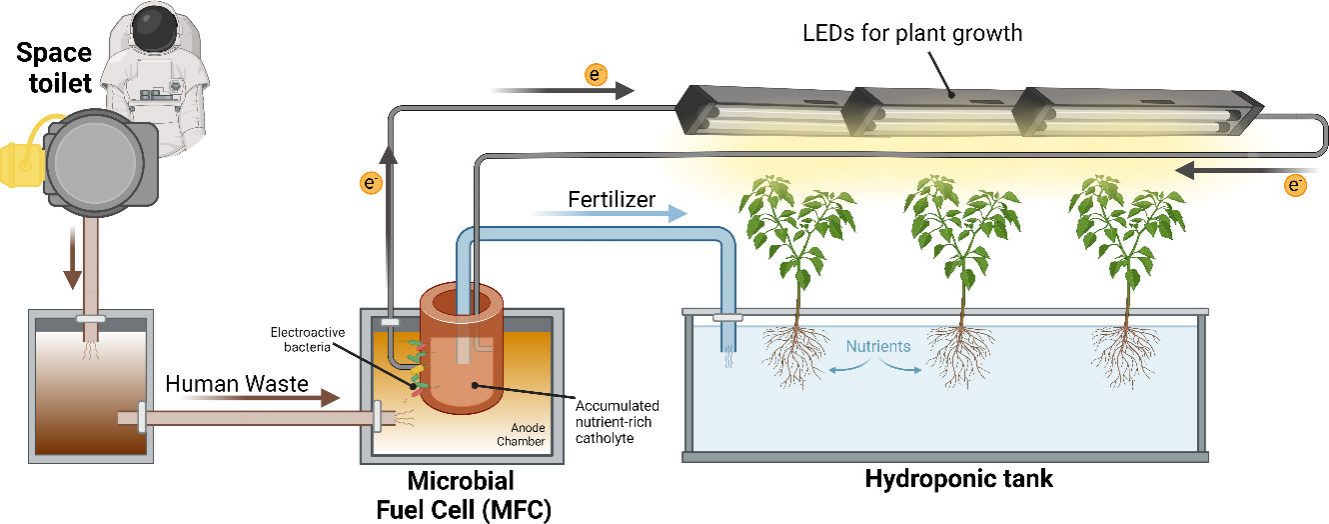
Concept diagram of a complete microbial fuel cell hydroponic (MFCH) system for space missions.

This concept, depicted in Figure 1, is being scaled up by integrating MFCs with hydroponics, as part of the EU-funded project ‘Microbial Hydroponics (Mi-Hy) grant no.: 101114746’, where electricity and nutrient-rich catholyte, produced by sewage-fed MFCs, are used to support plant growth in hydroponics along with a small percentage of synthetic fertilisers (unpublished data). Nutrient rebalancing and recycling using the Mi-Hy model can also be adopted in space stations, where human waste from astronauts can turn into electricity and liquid fertiliser for growing vegetables, in a controlled and pathogen-free manner.

Furthermore, the organic carbon (C) in a wastewater sample, which can be indirectly measured through biological oxygen demand (BOD), can be greatly reduced using MFCs. In an environment in which organic waste is metabolised or oxidised by microbes, the oxygen requirement to oxidise the organic waste will be proportional to the amount of organic matter present in the sample. The elemental balance of a feedstock like urine in the context of C and NPK has been previously described, demonstrating the correlation between organic and inorganic C energy into electricity [8]. MFCs have shown a BOD removal of more than 90%; for instance, a dual-chamber plant MFC has demonstrated a 93.16% BOD reduction [13]. In some cases, MFCs can even be used as a standalone BOD sensor [14–16].

In MFCs, the organic compounds can be degraded into carbon dioxide (CO_2_), hydrogen (H^+^) and electrons. In future MFCH systems, the CO_2_ released from the anodic activity could be redirected for plant uptake to enhance circularity and further increase the amount of ‘profitable’ C. In addition to this, the C is also being ‘stored’ as bacterial biomass [17], which helps reduce the BOD further.

## MFC addressing concerning factors in Space

Some of the potential concerns related to the use of MFCs for long-duration missions are listed below.

- Biofilm formation under zero- or micro-gravity: Spaceflight has been shown to increase the number of viable cells, biofilm biomass and thickness relative to normal gravity controls [18]. This could represent an improvement in the power output of the spaceflight MFCs due to better-developed surface-associated bacterial communities. Excessive biofilm biomass in MFCs relative to normal gravity could be managed using a thin anodic biofilm with higher power output when MFCs are operated at a low resistance load [19].
- Shear stress affecting the biofilm: Electroactive bacteria such as *Geobacter sulfurreducens* have been demonstrated to form matured biofilm under shear stress conditions (ranges from 1 Pa to 0.01 Pa), producing higher current with less time and increased metabolic rate, independent of their mass transport rate [20].
- Disinfectant/antimicrobial chemicals used in the toilet/urine flushing: Surfactants (e.g. sodium dodecyl sulphate) present in the hand wash/toilet cleaner used for toilet/urine flushing can be degraded by the anodic biofilm in MFCs (with an efficiency of 70%) [21,22]. Furthermore, the disinfectants are diluted with the flushed water, reducing their antimicrobial properties when fed to MFCs.
- Microorganisms developing extremophilic properties or antibiotic resistance: Studies on microbial communities found in the ISS did not show development of extremophilic or antibiotic-resistant communities, and hence, there is no direct reason for concern towards crew health [23]. Effective biofilms inside MFCs have nevertheless been shown to efficaciously suppress/kill pathogenic organisms and viruses, suggesting that the technology could act as a stopgap for when pathogens may enter the system [24,25].

## Conclusions

In order to achieve the ambitious goals of the future space exploration missions, a habitable environment should be equipped with efficient waste management and regenerative systems that allow nutrients to be recycled. MFC systems can be utilised in space explorations where micro-greens are growing as part of the astronauts’ living environment, which requires soil for growth, such as in the BEATRICE project [26]. This project aims to develop and test a prototype of an integrated bioregenerative system capable of recycling astronauts’ wastewater and generating fertiliser and nutrients with MFCs to produce edible vegetables, such as micro-tomatoes. MFC-generated liquid fertiliser could also be used to condition soil for better growth, as well as a terrestrial-MFC (TMFC, also known as soil MFC) module [27] integrated as part of a complete circular system. A TMFC is a variant of an MFC comprising two electrodes separated by a layer of soil, acting as the electrolyte, and connected externally by a wire or a load. These exemplars are used for soil remediation with concomitant bioelectricity production.

Using MFC systems for waste treatment in long-duration missions can help close the loop of ‘nutrients recycling’. The catalytic power of MFCs in technologies like Mi-Hy allows the production of vegetables in a self-sustainable manner, where the excessive C and other inorganic elemental nutrients (N, P, K) present in the urine/sewage can be turned into plant biomass and electricity. In a way, it is a system that can help carry, biotransform and recycle C in an otherwise C-deprived environment, without the need for additional C payload at the start of a mission. This can help overcome challenges in the current space waste treatment systems, such as resupply for consumables, generation of additional waste streams, nutrient loss in waste streams, and high temperature and pressure requirements.

## Abbreviations

BOD: biological oxygen demand
C: carbon
CO_2_: carbon dioxide
ISS: International Space Station
IWP: Ionomer-membrane Water Processor
MFC: microbial fuel cell
MFCH: MFC-hydroponic
Mi-Hy: Microbial Hydroponics
NPK: nitrogen, phosphorus and potassium
TMFC: terrestrial-MFC
